# Social determinants and adherence to treatment among Colombian women living with HIV/AIDS

**DOI:** 10.1186/1475-9276-11-S1-A1

**Published:** 2012-01-23

**Authors:** Marcela Arrivillaga

**Affiliations:** 1 Department of Public Health & Epidemiology, Pontificia Universidad Javeriana Cali, Cali, Colombia

## Background

There is a paucity of studies on the social determinants of adherence to antiretroviral therapy, especially in Latin American countries. The purpose of the study was twofold: to assess the relationship between antiretroviral adherence and social position among Colombian women with HIV/AIDS and to examine the possibility of expanding the medical concept of adherence to treatment including a “social determinants of health” perspective.

## Method

A mixed method approach with a qualitative and quantitative sequential design was applied. In the phase of formative research semi-structured interviews were conducted with 7 national experts in the field. The qualitative component of the study included 10 focus groups with a total of 99 women; in-depth interviews were conducted with 14 of these participants. Another 269 women from five different cities completed a socio-demographic and clinical questionnaire, an adherence to treatment questionnaire, and a social position survey designed according to the Colombian socioeconomic structure. Content analyses were applied to analyze the qualitative data and logistic regressions were used to analyze the quantitative data.

## Results

Significant statistical associations and qualitative patterns between adherence and social position were found. Women in a medium and high social position were more likely to present higher adherence behaviors than women in low social position. Also, healthcare system barriers, being a caregiver of children with HIV/AIDS, and individual coping styles were found as critical factors for adherence behaviors.

## Conclusions

Adherence to treatment in Colombian HIV positive women is determined by their social position. Research on antiretroviral adherence and the concept of adherence itself should include a “social determinants of health” perspective in order to maximize the likelihood of obtaining better clinical outcomes. The findings of the study should also serve as a point of reference for reviewing current healthcare guidelines for people living with HIV/AIDS in Colombia.

